# Safety and Efficacy of Single-Stage Synchronous Bilateral VATS Talc Poudrage for Malignant Pleural Effusion

**DOI:** 10.3390/cancers18111676

**Published:** 2026-05-22

**Authors:** Antonio Mazzella, Sara Degiovanni, Elena Mariani, Giorgia Cerretani, Luca Bertolaccini, Monica Casiraghi, Giulia Sedda, Giorgio Lo Iacono, Lorenzo Spaggiari

**Affiliations:** 1 Division of Thoracic Surgery, IEO European Institute of Oncology IRCCS, 20141 Milan, Italy; sara.degiovanni@ieo.it (S.D.); elena.mariani@ieo.it (E.M.); giorgia.cerretani@ieo.it (G.C.); luca.bertolaccini@unimi.it (L.B.); monica.casiraghi@ieo.it (M.C.); giulia.sedda@ieo.it (G.S.); giorgio.loiacono@ieo.it (G.L.I.); lorenzo.spaggiari@ieo.it (L.S.); 2 Department of Oncology and Haemato-Oncology, University of Milan, 20141 Milan, Italy

**Keywords:** lung cancer, pleural effusions, VATS, talc poudrage, pleurodesis, bilateral talc poudrage

## Abstract

**Backgrounds/Objectives**: Malignant pleural effusion (MPE) is a frequent complication of advanced cancer, and talc pleurodesis via video-assisted thoracoscopic surgery (VATS) represents a standard palliative treatment with high efficacy. However, evidence regarding synchronous bilateral pleurodesis in patients with bilateral MPE is limited. This study evaluates the feasibility, safety, and outcomes of a single-stage bilateral VATS talc pleurodesis approach. **Materials and Methods**: We retrospectively analyzed patients undergoing synchronous bilateral VATS talc poudrage between 2000 and 2025 at a single tertiary cancer center. Inclusion criteria included adult patients with bilateral MPE, expandable lungs, and suitability for surgery. Preoperative assessment involved imaging and multidisciplinary evaluation. Perioperative data, complications, mortality, and recurrence rates at 30 days and 3 months were collected. Survival and pleural effusion-free survival were estimated using the Kaplan–Meier method. **Results**: Thirty patients were included (median age 63.2 years). The most common primary tumors were breast (43%), lung (30%), and ovarian cancer (17%). Mean operative time was 78.6 min, with no intraoperative complications. Mean hospital stay was 6 days. Postoperative morbidity included atrial fibrillation (13%) and respiratory failure (6.6%), both managed conservatively. Thirty-day mortality was 3%. Pleural effusion recurrence occurred in 6.6% at 3 months and 10% at 7 months. Mean follow-up was 9.7 months. **Conclusions**: Synchronous bilateral VATS talc pleurodesis is a feasible and safe procedure in selected patients with bilateral MPE with acceptable morbidity. Further prospective studies are needed to confirm these findings and refine patient selection.

## Simple Summary

This study was conducted to address a clinical gap in the management of patients with bilateral malignant pleural effusion (MPE), a condition that significantly impairs quality of life in advanced cancer. While talc pleurodesis via video-assisted thoracic surgery (VATS) is a well-established treatment for unilateral disease, there is limited evidence on the safety and feasibility of performing the procedure simultaneously on both sides. We aimed to demonstrate that synchronous bilateral talc pleurodesis can be safely performed in selected patients, providing effective symptom control without increasing perioperative risks. By analyzing clinical outcomes, complication rates, and recurrence, we sought to evaluate whether this approach could represent a valid alternative to staged procedures. This research may have an important impact on clinical practice by reducing the number of hospital admissions, shortening overall treatment time, and improving patient comfort. Ultimately, it supports a more efficient, patient-centered strategy in the palliative care of individuals with advanced malignancies.

## 1. Introduction

Malignant pleural effusion (MPE) is a common complication of advanced malignancies, occurring in approximately 10–15% of patients with cancer and representing a significant cause of morbidity and healthcare utilization [1,2]. It is most frequently associated with lung cancer, breast cancer, and lymphomas, although virtually any malignancy can involve the pleura, with or without a preoperative or intraoperative diagnosis, linked to tumoral cellules or specific tumoral markers in the liquid [3,4]. The presence of MPE generally reflects advanced-stage disease and is associated with poor prognosis, with low median survival, depending on tumor type and patient characteristics [2,5].

The primary objective in the management of MPE is palliative, focusing on symptom relief—particularly dyspnea—to ensure the best quality of life for the patient for starting or continuing systemic therapies [6]. Available treatment options include repeated thoracentesis, indwelling pleural catheters (IPCs), and chemical pleurodesis [7,8]. Among these, surgical talc pleurodesis (via VATS, associated with pleural biopsies) remains one of the most widely used and effective strategies for achieving durable control of pleural effusion [9,10].

The intrapleural instillation of talc triggers a robust chemical pleuritis characterized by an initial exudative phase, driven by the activation of mesothelial cells and the release of pro-inflammatory cytokines such as IL-8 and MCP-1. This physical-chemical interaction promotes the recruitment of macrophages and neutrophils, leading to a fibrin-based adhesion process that eventually matures into extensive pleural fibrosis, effectively obliterating the pleural space and preventing further fluid accumulation [10,11]. It can be administered either as slurry through a chest drain or as poudrage during thoracoscopy. Several randomized controlled trials and meta-analyses have demonstrated that talc pleurodesis provides high success rates, ranging between 85% and 95% and is superior to other sclerosing agents such as bleomycin or tetracycline derivatives [11,12,13,14]. As widely demonstrated, surgical thoracoscopic talc poudrage is the gold standard treatment for different reasons; first of all it is associated with a direct visualization of the pleural cavity for evaluating the lung’s capacity for re-expansion; it allows an uniform talc distribution everywhere in the pleural cavity; with a correct pleural fluid postoperative drainage. For these reasons, it is associated with higher efficacy in selected patients [13,14,15].

Despite its effectiveness, talc pleurodesis is not without risks. Common adverse events include chest pain and fever, while rare but serious complications such as acute respiratory distress syndrome (ARDS) have been reported, particularly in association with small-particle talc [16,17]. Current guidelines recommend the use of graded talc to minimize systemic inflammation and pulmonary complications [6,18].

The safety and efficacy of talc pleurodesis are strictly dependent on particle size distribution; the use of calibrated, large-particle talc (mean diameter > 15 µm) is essential to minimize systemic dissemination. Conversely, small-particle talc can migrate from the pleural space through the lymphatic stomata into the systemic circulation, potentially triggering a sequestered inflammatory response in the pulmonary vasculature and increasing the risk of acute respiratory distress syndrome (ARDS) [18,19].

A significant proportion of patients with MPE present with bilateral pleural involvement, posing additional therapeutic challenges. Standard management in these cases typically involves staged unilateral interventions, which may result in prolonged hospitalization, repeated exposure to anesthesia, and delayed symptom control [7,14,20,21,22,23]. In a population with limited life expectancy, minimizing treatment burden is of paramount importance.

To date, there is a paucity of data regarding the feasibility and safety of synchronous bilateral talc pleurodesis. Concerns about potential respiratory compromise and increased perioperative risk have limited the adoption of this approach, and current international guidelines do not provide specific recommendations on bilateral management strategies [6,22].

In this context, the present study aims to evaluate the feasibility, safety, and clinical outcomes of synchronous bilateral talc pleurodesis in patients with malignant pleural effusion, potentially offering a more efficient and patient-centered therapeutic approach.

## 2. Materials and Methods

We retrospectively observed pre-, peri-, and postoperative data of patients undergoing VATS pleural biopsies and talc poudrage 2000 to 2025 at the European Institute of Oncology (IEO) in order to definitively treat malignant pleural effusion resulting from metastasis linked to other cancers. We selected only patients underwent synchronous bilateral VATS talc poudrage.

The patients’ medical and operative records were reviewed in terms of original intervention, time between the first surgery and possible relapse, intra and postoperative comorbidities, mortality and consequent follow-up. The study was conducted in accordance with the Declaration of Helsinki and is reported in accordance with the STROBE (STrengthening the Reporting of OBservational studies in Epidemiology) guidelines. The study was approved by the Ethics Committee of the European Institute of Oncology (UID 5221, 19 November 2025). Written informed consent to undergo the procedure and to use clinical and imaging data for scientific or educational purposes, or both, was obtained from all patients before the operation.

Inclusion criteria were: patients older than 18 years with bilateral pleural effusion; patients with oncologic disease of any type; patients deemed suitable for talc pleurodesis (i.e., with expandable lung following pleural fluid evacuation); and patients undergoing bilateral pleural biopsies and talc pleurodesis in the same surgical session.

Patients who underwent synchronous bilateral video-assisted thoracoscopic pleural biopsies and talc pleurodesis from 2000 to the present were included. The analyzed data comprised demographic characteristics of the cohort, patient symptoms at referral, and surgical details (operative time, technique, and surgical approach); perioperative and postoperative complications; and surgical outcomes at follow-up. In particular, we evaluated recurrence of pleural effusion (unilateral or bilateral) at 30 days and 3 months after surgery, as well as any subsequent need for treatment.

### 2.1. Pre-Operative Management

Pre-operative staging consisted of a total body computed tomography scan (CT scan) and a positron emission tomography (PET) with fluorodeoxyglucose (FDG), an accurate preoperative cardiological and respiratory assessment in order to evaluate and minimize anesthesiologic issues during the intervention (including echocardiography).

All patients were subsequently reviewed by a multidisciplinary team, in collaboration with oncology specialists, to confirm the indication for surgery.

In cases of massive preoperative pleural effusion, an 8Fr drain was preferably placed (either unilaterally or bilaterally) on the day prior to surgery. This was performed to drain the fluid, promote controlled lung re-expansion (or assess re-expansion capacity), and facilitate anesthetic maneuvers during induction.

### 2.2. Exclusion Criteria

Patients with a history of cardiac disease or uncompensated chronic heart failure were excluded from the study and were candidates for sequential unilateral thoracoscopy.

Similarly, patients presenting with trapped lung—characterized by the inability of the lung to re-expand following drainage—were clearly excluded from the procedure.

Furthermore, patients with poor performance status or an oncological prognosis of less than three months were strictly excluded.

### 2.3. Surgical Technique

Surgical procedures were performed under general anesthesia and by double lumen intubation, lateral decubitus positions. The surgical approach typically prioritized the side with the more significant pleural effusion. Lung isolation was achieved via a double-lumen endobronchial tube to allow for unilateral collapse during the procedure. Following the completion of the first stage, bilateral ventilation was restored using lung-protective strategies—specifically maintaining a maximum PEEP of 3 cmH_2_O to mitigate the risk of re-expansion pulmonary edema. Finally, the contralateral lung was isolated to proceed with the second stage of the intervention.

In some cases, when pleural effusions were associated with symptomatic pericardial effusions linked to neoplasm, this intervention, was associated with pericardial definitive drainage (pericardial–peritoneal window) as already described in our previous paper [24].

**Figure 1:**
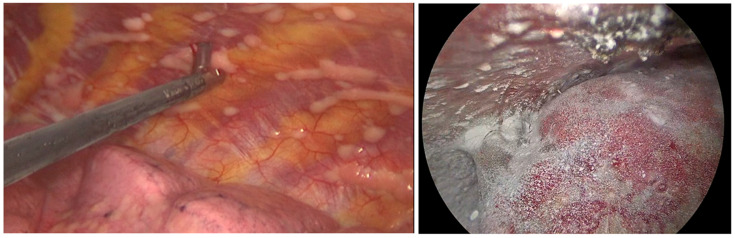
On the **left**, VATS pleural biopsies by bioptic forceps in pleural carcinosis. On the **right**, injection of talc into pleural cavity.

Two thoracoscopic ports of approximately 1 cm each were created in the fifth and seventh/eighth intercostal spaces. A 10 mm thoracoscope was then introduced into the pleural cavity. After pleural fluid aspiration and thorough exploration of the entire pleural space, abnormal parietal or visceral pleura areas were identified and biopsied. A frozen section exam was performed in order to obtain a preliminary diagnosis and the correct specimen adequacy for the definitive diagnosis. In the absence of macroscopically suspicious areas, random biopsies were performed. Lung re-expansion is also assessed intraoperatively using a ventilation test. In case of positive frozen section and satisfactory lung re-expansion, talc pleurodesis was performed by insufflating 8 g of asbestos-free sterile talc everywhere in the pleural cavity (Figure 1). At the end of procedure, in order to correctly drain pleural cavity, two chest drains were then placed through the surgical ports, on for the apical, the second for the basal part.

After completion of the procedure on the first side, an identical protocol was followed for the contralateral side.

Chest drains were maintained at a continuous suction off (−15 cmH_2_O). Drains were removed on the fourth postoperative day or when daily output fell below 300 mL should they have remained in place for a longer duration.

### 2.4. Statistical Methods

Continuous variables were expressed as mean ± standard deviation (SD) and compared using Student’s *t*-test or the Mann–Whitney U test, as appropriate based on data distribution. Categorical variables were presented as absolute frequencies and percentages, and the association with pleural effusion recurrence was assessed using Fisher’s exact test, which is preferred for small sample sizes with low expected cell frequencies. A *p*-value < 0.05 was considered statistically significant. The analyses were performed using SAS software version 9.4 (Cary, NC, USA). All *p*-values were two-sided.

Postoperative death was defined as 30-day mortality or longer if mortality occurred during hospitalization. Survival time for patients still alive at the last follow-up date was considered censored.

We considered as recurrence, the presence of symptomatic pleural effusions after surgery, diagnosed by X-ray chest or CT scan, determining a relevant clinical impact. Cumulative incidence of relapse after intervention was estimated using the cumulative incidence function with death considered as a competing risk event. Patients who were still alive were censored at the date of the last follow-up. Overall survival was plotted using the Kaplan–Meier method. All statistical analyses were performed using SAS software version 9.4 (Cary, NC, USA). Generative artificial intelligence (Gemini Google) has been used in this paper to generate Kaplan–Meier graphics and the authors have reviewed and edited the output and take full responsibility for the content.

## 3. Results

From 2000 to the present, 36 patients underwent VATS synchronous bilateral talc pleurodesis. Six of them refused to consent to the processing of data. Then we analyzed 30 patients. Demographics and preoperative outcomes are shown in Table 1. Intraoperative and postoperative outcomes are shown in Table 2.

The median age was 63.2 years (IQR: 51.75–67.75), with no gender predominance (M:F = 16:14). The demographic and preoperative characteristics are presented in Table 1.

The majority of patients (22 out of 30 patients—73%) presented with dyspnea; 10 patients (33%) reported cough, and 6 (20%) reported chest pain. A minority of patients (5 out 30—16%) were asymptomatic, and pleural effusion was detected incidentally during radiological follow-up for a previously diagnosed malignancy.

Regarding the primary malignancies associated with bilateral pleural carcinomatosis, the underlying origins were breast cancer in 13 cases (43.3%), ovarian cancer in 5 (16.6%), lung cancer in 9 (30%), renal cancer in 1 (3.3%), and gastric cancer in 2 cases (6.6%).

In four out of 30 cases, the bilateral procedure was combined with the creation of a pericardio-peritoneal window due to the concomitant presence of symptomatic pericardial effusion.

The mean length of hospital stay was 6 days (range [5–15] days).

All surgical procedures were performed using video-assisted thoracoscopy. The mean operative time was (88.4 ± 35.2) minutes. No intraoperative complications were recorded. No intraoperative mortality or morbidity was reported.

Regarding 30-day postoperative morbidity, four patients experienced episodes of atrial fibrillation, which resolved following amiodarone administration. Additionally, two cases of respiratory failure occurred, successfully managed with diuretic therapy and CPAP.

The 30-day mortality rate was 3% (one patient deceased due to cardio-respiratory failure).

**Figure 2:**
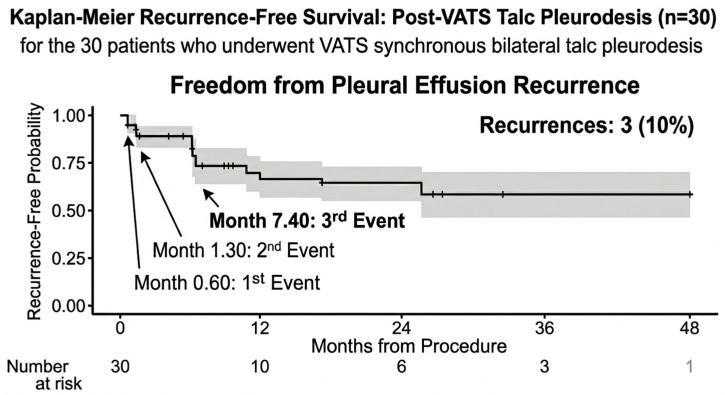
Recurrence-free survival of pleural effusions.

The 3-month recurrence rate of pleural effusion was 6.6% (2 cases, 1 unilateral and 1 bilateral), increasing to 3 cases after 7 months (10%); all such cases were managed with the re-insertion of a pleural drain (Figure 2).

Mean follow-up was 9.7 months (range [0–55] months—follow-up data also accounted for the 30-day mortality case).

No statistically significant predictors of recurrence were identified.

## 4. Discussion

In the present study, we assessed the feasibility and clinical outcomes of synchronous bilateral talc pleurodesis in patients with bilateral 54 malignant pleural effusion. Our findings suggest that this approach is technically feasible and can be performed with an acceptable safety profile in carefully selected patients.

Talc pleurodesis remains a cornerstone in the management of MPE, with robust evidence supporting its efficacy in preventing recurrence and improving symptoms [9,12,13,14,15,16,17]. Large randomized trials and meta-analyses have consistently demonstrated high rates of successful pleurodesis, particularly when talc is administered via thoracoscopy [16,17,18,19,20].

The superior efficacy of VATS talc pleurodesis, compared to other pleurodesis methods, is now an acquired and well-structured notion in the literature [10,11,12] and it represents the gold standard in the palliative treatment of MPE. The reasons for the success rate (85–95%) of this technique are represented by the complete and direct visualization of the pleural cavity before and after performing pleural biopsies, the live visualization of lung re-ventilation during surgery and the possibility to spread and distribute the talc everywhere into pleural cavity. In addition, this technique allows to place correctly the chest drains, in order to obtain the complete dryness of chest cavity during post-operative period; this aspect facilitates and catalyzes the uniform inflammatory and sclerotic process underlying pleurodesis [14,15,16,20].

However, the vast majority of these studies focus on unilateral disease, and evidence regarding bilateral pleural involvement remains limited.

The rationale for a synchronous bilateral approach lies in its potential to reduce overall treatment burden. Patients with advanced malignancies often have limited functional reserve, and repeated procedures may negatively impact both quality of life and clinical outcomes [5,7]. Sequential unilateral pleurodesis requires multiple hospital admissions or prolonged hospitalization, as well as repeated exposure to anesthesia. In contrast, a single-stage bilateral intervention may provide faster symptom relief and streamline care delivery.

From a physiological standpoint, the main concern regarding bilateral pleurodesis is the potential for respiratory compromise. Talc induces a diffuse inflammatory response within the pleural space, which could theoretically impair gas exchange when performed bilaterally [14,18,20,21]. Historical reports of ARDS following talc pleurodesis have raised concerns about systemic dissemination of talc particles, particularly with non-calibrated formulations [18,19,20,21]. However, the use of graded talc has significantly improved the safety profile of the procedure, reducing the incidence of severe pulmonary complications [19,21].

In our series, the observed complication rates appear comparable to those reported in the literature for unilateral pleurodesis, where common adverse events include chest pain, fever, and transient inflammatory responses or common complications generally associated with thoracic surgery, such as atrial fibrillation [17,20]. The two cases of respiratory failure, likely attributable to postoperative re-expansion pulmonary edema, resolved completely with medical therapy and CPAP, without requiring invasive mechanical ventilation. Furthermore, the 30-day mortality rate of 3% (n = 1) is consistent with current literature, which reports rates ranging from 2% to 6% for unilateral VATS talc poudrage in patients with malignant pleural effusion [21]. Even if, in other reports no 30-day mortality was observed [21], it must be clarified that the single mortality in our series involved a patient presenting with concomitant bilateral pleural effusion and pericardial effusion. While rigorous patient selection is paramount, these individuals inherently face a higher perioperative risk compared to those undergoing unilateral intervention. Importantly, no significant increase in severe complications was observed, suggesting that bilateral treatment may be safely considered in selected patients with adequate respiratory reserve.

The 3 and 7-month recurrence rate in our series was 6.6 and 10% respectively (3/30 cases), which was successfully managed with re-insertion of a pleural drain. This result compares favorably with existing literature; for instance, other authors reported recurrence rates for VATS talc poudrage ranging from 10% to 15% [7,8,25,26,27,28,29,30], confirming the high efficacy of this technique in ensuring long-term pleural symphysis compared to other methods.

An important aspect to consider is the evolving role of indwelling pleural catheters. Randomized trials, such as the TIME2 study, have demonstrated that IPCs provide effective symptom control and may reduce hospitalization compared to talc pleurodesis [25,27,28,29]. However, IPCs require ongoing management and are associated with potential complications such as infection, catheter blockage, and patient discomfort. Moreover, spontaneous pleurodesis rates with IPCs are variable and may not occur in all patients [27,28,29]. In contrast, talc pleurodesis offers a time-limited intervention with the potential for definitive control of effusion.

From a healthcare system perspective, synchronous bilateral pleurodesis may also have economic advantages. By reducing the number of procedures, hospital admissions, and overall length of stay, this approach could potentially lower healthcare costs and optimize resource utilization. Although cost-effectiveness was not directly evaluated in this study, previous analyses have highlighted the economic impact of different MPE management strategies [30].

We did not deliberately investigate overall survival or disease-free survival, primarily because these are beyond the scope of this paper. Furthermore, our data encompass various tumor histotypes that are markedly different from one another; analyzing such data would risk creating confusion and detracting from the main focus of this study, which is the feasibility and outcomes of this surgical procedure, regardless of the primary tumor type.

The present study has several limitations. First, the relatively small sample size limits the generalizability of the findings. Second, the lack of a control group prevents direct comparison with staged unilateral procedures or alternative treatment strategies. Third, the potential for selection bias must be acknowledged, as patients undergoing bilateral pleurodesis are likely to represent a subgroup with better performance status and preserved pulmonary function. Additionally, long-term outcomes and patient-reported quality-of-life measures were not systematically assessed. In addition, We did not compare the outcomes of over 3000 standard unilateral talc procedures effected in out Institution in the period, given the small number of bilateral talc procedures. This recommendation is intended only for highly selected patients. Lastly, the 26-year study period was characterized by substantial evolution in clinical practice, such as changes in talc formulation, surgical and anesthetic techniques or the advent of modern systemic treatments.

Despite these limitations, our study addresses an important gap in the current literature. To our knowledge, data on synchronous bilateral talc pleurodesis remain scarce, and our findings contribute to the growing interest in more efficient and patient-centered approaches to MPE management.

Future prospective studies with larger cohorts and comparative designs are warranted to confirm these findings and to better define patient selection criteria. In particular, the integration of clinical, radiological, and functional parameters may help identify patients who are most likely to benefit from a bilateral approach.

## 5. Conclusions

In conclusion, synchronous bilateral talc pleurodesis appears to be a feasible and safe option in selected patients with bilateral malignant pleural effusion. This strategy may reduce treatment burden and improve the efficiency of care without compromising clinical outcomes, representing a promising alternative to traditional staged approaches.

## Figures and Tables

**Table 1:** Demographics and preoperative outcomes.

Variable	Total (n = 30)	No Recurrence (n = 27)	Recurrence (n = 3)	*p*-Value
Gender (M/F)	16 M/14 F	14 M/12 F	1 M/2 F	1.000
Age (years)	63.2 ± 11.8	62.8 ± 12.1	65.5 ± 10.9	0.678
BMI (kg/m^2^)	25.9 ± 3.6	26.0 ± 3.8	24.9 ± 2.4	0.584
ASA				0.812
2 (n, %)	19 (63.3%)	17 (65.4%)	1 (33.0%)	
3 (n, %)	11 (36.7%)	9 (34.6%)	2 (66.0%)	
Clinical symptoms				0.518
Dyspnea	22 (73.3%)	20 (76.9%)	2 (66.0%)	
Pain	6 (20%)	5 (16.6%)	1 (33.0%)	
Cough	10 (33%)	10 (33%)	0 (0.0%)	
Asymptomatic	5 (16.6%)	5 (18.5%)	0 (0.0%)	

**Table 2:** Intra and postoperative outcomes.

Variable	Total (n = 30)	No Recurrence (n = 27)	Recurrence (n = 3)	*p*-Value
Operative Time (min)	88.4 ± 35.2	86.5 ± 36.1	100.3 ± 29.5	0.468
Primary Malignancy:				0.452
Breast	13 (43%)	12 (44%)	1 (25.0%)	
Ovarian cancer	5 (16.6%)	5 (18.5%)	0 (0%)	
Lung cancer	9 (30%)	7 (25.9%)	2 (50.0%)	
Renal cancer	1 (3.3%)	1 (3.2%)	0 (0.0%)	
Gastric cancer	2 (6.6%)	2 (7.4%)	0 (0.0%)	
Surgical procedure				0.551
Bilateral VATS	26 (86.7%)	24 (88.8%)	2 (66.6%)	
VATS + peric. window	4 (14.8%)	3 (11.1%)	1 (33.3%)	
Length of Stay (d)	7.6 ± 3.4	7.5 ± 3.6	8.3 ± 2.1	0.665
Chest Tube Duration (d)	5.3 ± 2.1	5.2 ± 2.2	5.8 ± 1.3	0.598
Complications:				0.142
YES (n, %)	6 (20.0%)	4 (15.4%)	2 (66.6%)	
NO (n, %)	24 (80.0%)	23 (84.6%)	1 (33.3%)	

## Data Availability

The original contributions presented in this study are included in the article. Further inquiries can be directed to the corresponding author(s).
